# Medical Items

**Published:** 1896-02

**Authors:** 


### *MEDICAL ITEMS.*

Eight extra pages in this issue.

Dr. A. H. Van Dyke, late House Surgeon at the Hospital for the Ruptured and Crippled, New York City, has returned to Atlanta to practice his profession.

Dr. C. M. Drake, late Surgeon to the Western Division of the Southern Railway at Knoxville, has been appointed Chief Surgeon to the Southern System, with headquarters in Atlanta.

Dr. George Dock, at present Professor of Practice of Medicine and Pathology in the University of Michigan, Ann Arbor, has been elected Professor of Pathology and Bacteriology in the Jefferson Medical College.

Dr. Jas. B. Baird has been elected Professor of Practice in the Southern Medical College, in place of Dr. C. S. Webb resigned. Dr. J. C. Olmsted is appointed Dr. Baird's successor on the State Examining Board. These are both excellent selections.

Following are the officers of the Atlanta Society of Medicine for the current year: President, Dr. R. R. Kime; Vice-President, Dr. W. A. Crow; Secretary, Dr. W. L. Champion; Treasurer, Dr. L. B. Grandy; Corresponding Secretary, Dr. Dunbar Roy.

The *Western Reserve Medical Journal* has retired from the field and is succeeded by the Cleveland (Ohio) *Journal of Medicine.* The latter will be the official journal of the Cleveland Medical Society, and will be edited by Dr. Henry I. Upson. The new journal has our best wishes for success.

A MEDICAL gentleman always recognizes that it takes money to publish a good journal. He does not fly into a passion when he receives his reminder in the form of a bill for subscription, but as he hopes that his patrons will pay him, he promptly remits the amount to his journal, *tuto, cito et jucunde.—Ex.*

---

DR. J. E. GILLESPIE, a prominent physician, of Chipley, Georgia, died suddenly from some heart trouble January 6th. Dr. Gillespie was apparently in his accustomed health and had been attending to his professional duties up to the hour of his death. He formerly practiced in Columbus, where he was city physician for a number of years.

---

PHYSICIANS generally will congratulate the *Ladies' Home Journal* upon their late decision in declining to publish in the future any advertisement of a "medical, remedial, or curative nature," upon the ground that it is not willing to have even the appearance of vouching for such advertisements. This decision is somewhat bold, but very commendable, and other publications would do well to adopt it.

---

DR. JOHN S. BILLINGS, late Deputy Surgeon-General U. S. Army, on the evening of November 30th, was presented with a check for $10,000 duly inclosed in a silver box, bearing the following inscription: "From 259 physicians of the United States and Great Britain, in grateful recognition of services to medical scholars." The services here alluded to was the completion of the Index Catalogue of the Library of the Surgeon-General's office, Washington.

---

THE patient had just described his symptoms, and the physician grasped him by the hand. "My dear fellow," he cried. "I cannot tell you how delighted I am you should have come to me. You have a disease which has baffled the profession for years. Hitherto it has always proved fatal, and I've always wished to experiment on it myself. If I save you I shall be immortal, and if I don't—what's the odds?"—*Harper's Bazar.*

---

BAILY & FAIRCHILD CO., of New York, take pleasure in announcing to the medical profession the establishment of the Doctor's Story Series, to be issued quarterly at $2.00 a year, 50 cents a number. Each number will consist of a complete work of fiction by medical authors. Only such works as are of established value will be reproduced in this popular form. King's "Stories of a Country Doctor" will be issued January, 1896, to be followed in March by Dr. Phillips' wonderful novel, "Miskel," and later by a new novel now in preparation by the same author.

---

THE three Examining Boards in 1895 examined and licensed one hundred and twenty-five applicants. One hundred and five were regulars, thirteen eclectics, and seven homeopaths. It is said to have been the policy of the boards not to be too severe in their examinations all at once. It is expected that they will be more rigid in the future. We hope that the boards will see that the spirit and letter of the law are fully carried out. Its friends look to it as the only means of protecting our people from hordes of medical tramps and numbers of incompetent graduates. The law is a good one and *deserves to be carried out.*

---

WE have received from Dr. W. O'Daniel, ex-Principal Physician to the Penitentiary, an interesting paper entitled "Vital Statistics, and the Hygiene of the Hospitals and Prison Camps of the Georgia Penitentiary from October 1, 1890, to October 1, 1894." This is an elaboration of a paper published in this JOURNAL, August, 1895. The data presented by Dr. O'Daniel show the prison camps to have been in excellent sanitary condition during this period, the death-rate from preventable causes being much lower than one would probably expect. During the last six months of Dr. O'Daniel's service, out of a prison population of 2,652, there were only fifteen deaths from all causes. This represents a yearly death-rate of only 11.5 per thousand or a fraction over one per cent.

---

AT a late banquet of the Denver Clinical Society, the following toast was drunk amid great applause:

Here's to the man who loves his wife,
And loves his wife alone;
For many a man loves another man's wife
Far better than his own.

Here's to the man who rocks his babe,
And rocks his babe alone;
For many a man rocks another man's babe,
Yet thinks it is his own.

—*Colorado Medical Journal.*

Here's to the health of wife and babe
Who stay at home alone,
While the husband is away with the other man's wife
Rather than be with his own.

---

THE ATLANTA CONSTITUTION of January 19th contains an interesting article by Mr. Frank Weldon on Dr. Crawford W. Long's early use of ether. The article publishes for the first time a letter from Dr. Long to a friend in Athens in which he ordered the ether used on the occasion of his first operation. A monument to Dr. Long is urged, and the statement is made that the Messrs. Venable, of Atlanta, nephews of the first patient operated upon, will contribute the granite, and the Georgia Railroad will give transportation. Dr. Hardman, of Harmony Grove, has volunteered $500, and Dr. Bosworth, of Atlanta, $100 toward a monument. This seems to be a very fair start. The JOURNAL would like to see the Georgia doctors take the matter up and erect the proposed monument to Dr. Long. We will be glad to publish any communications from our readers on the subject.

---

WHAT is the matter with medical journalism in Philadelphia? A few months ago the *Medical and Surgical Reporter* removed to New York, though it soon returned to Philadelphia. The editorial office of the *Times and Register* has been transferred from Philadelphia to Boston. And the *Medical News*, which, for more than thirty years, has been one of the leading medical periodicals of Philadelphia, as well as of the country generally, removed its editorial and publication offices to New York the first of the year. We can only surmise what were the "business reasons" that made this move advisable, and we surely hope that the *News* will find in its new home that encouragement and support which it so richly deserves. Under the late management of Dr. Gould the *News* had been brought to a degree of excellence never before attained, and it had become a high-toned and independent scientific medical weekly, standing only for what was honorable and ethical in medicine, which the profession in Philadelphia could not well afford to do without. The *News* has our best wishes wherever it goes.

---

THE World's Congress of Medico-Climatology will hold a National Convention in San Antonio, Texas, February 20th, continuing three days. The object of the congress is to make a thorough, careful, scientific, and systematic classification of the climates and resorts of the world, and particularly of the United States, as regards their therapeutical value in all forms of disease. Also to examine into the merits of mineral waters and properly classify them. It is well known by the medical profession that up to the present time no reliable scientific data are obtainable. This work is to be done under the auspices and direction of the profession, through their regularly organized societies acting in co-operation with the congress. The membership is composed of ten (10) representatives appointed by the secretary or proper officer of each of the various State medical societies, and of physicians in good standing in the profession, who are passed upon favorably by the Committee on Membership. Members of the profession are cordially invited to attend. The President of the Congress is Dr. J. A. Robison, of Chicago; the Secretary, Dr. W. S. Rowley, San Antonio, Texas.

---

FOLLOWING are some United States Court decisions relative to subscriptions to periodicals. Read them:

1. Subscribers who do not give express notice to the contrary are considered as wishing to redew their subscriptions.

2. If subscribers order the discontinuance of their periodicals, the publisher may continue to send them until all arrearages are paid.

3. If subscribers neglect or refuse to take their periodicals from the post-office to which they are directed, they are responsible until they have settled their bills and ordered them discontinued.

4. If subscribers move to other places without informing the publisher, and the papers are sent to the former address, they are held responsible.

5. The courts have decided that refusing to take periodicals from the office, or removing and leaving them uncalled for, is *prima facie* evidence of intentional fraud.

6. If subscribers pay in advance they are bound to give notice at the end of the time if they do not wish to continue taking it; otherwise the publisher is authorized to send it and the subscriber will be responsible until an express notice, with payment of all arrearages, is sent to the publisher.

The latest postal laws are such that newspaper publishers can arrest any one for fraud who takes a paper and refuses to pay for it. Under this law the man who allows his subscription to run along for some time unpaid, and then orders it discontinued, or orders the postmaster to mark it "refused," and have a postal card sent notifying the publisher, leaves himself liable to arrest and fine the same as for theft.

